# Coupling coordination relationship between geology–geomorphology and ecology in Northeast China

**DOI:** 10.1371/journal.pone.0266392

**Published:** 2022-04-07

**Authors:** Guofang Tao, Qigang Jiang, Chao Shi, Chaoqun Chen, Zhaoheng Jiang

**Affiliations:** 1 College of Geo–Exploration Science and Technology, Jilin University, Changchun, China; 2 Department of History and Geography, Tonghua Normal University, Tonghua, China; 3 North Automatic Control Technology Institute, Taiyuan, China; 4 Shenyang Center of China Geological Survey, Shenyang, China; 5 Key Laboratory for Evolution and Ecological Effect in Black Land of China Geological Survey, Shenyang, China; University of Waikato, NEW ZEALAND

## Abstract

Northeast China is an important ecological barrier and commodity grain base in China. The coupling coordination relationship between geology–geomorphology and ecology has become a critical background condition for ecosystem protection and sustainable development. Taking Northeast China as a case (accounting for about 13% of China’s land area), 9 divisions are divided according to the characteristics of regional ecology and geology–geomorphology, and 17 indicators are selected to build an evaluation index system. Methods of analytic hierarchy process, entropy weight and game theory are used to determine the index weights. Based on the coupling coordination degree (CCD) model, the spatial coupling coordination characteristics of geology–geomorphology and ecology are studied. The variation characteristics of the Normalized Difference Vegetation Index (NDVI) are evaluated by Sen+Mann–Kendall (Sen+MK) method. Our results are as follows. (1) The coupling between geology–geomorphology and ecology is strong, but the spatial differentiation of CCD is obvious. Nine divisions are evaluated as two high–level, three medium–level and three low–level coordination types and one mild imbalance type. (2) The plain divisions Ⅰ and Ⅳ where the typical black soil belt is located are high coordination types. Restricted by geology–geomorphological conditions or ecological conditions, mountain divisions Ⅲ and Ⅶ and plain division Ⅴ are moderate coordination types, mountain divisions Ⅱ and Ⅷ and plateau division Ⅸ are low coordination types, and mountain division Ⅵ is mild imbalance type. (3) The variation trend of NDVI shows a significant increase in divisions Ⅲ, Ⅴ, Ⅰ, Ⅱ and Ⅶ. it shows a significant decrease in part of divisions Ⅳ, Ⅵ, Ⅷ and Ⅸ, and ecological management and construction should be strengthened in these divisions. The research shows that the CCD model method is feasible for evaluating the relationship between geology–geomorphology and ecology and can provide eco–geological background information for Northeast China.

## Introduction

Geological and geomorphological conditions affect the spatiotemporal distribution pattern of vegetation and ecology at the regional scale. In contrast to the significance and multi–periodicity of climate change, the characteristics of geology and geomorphology are relatively stable. It takes at least tens of thousands to hundreds of thousands of years to form a specific spatial distribution pattern of geology–geomorphology [1,2]. The spatial differentiation of geology–geomorphology leads to the redistribution of zonal water and heat conditions, which directly or indirectly affect the regional ecological process and then alter changes in the structure and function of ecosystems [3]. Northeast China area is a relatively complete natural geographical region, which is an important forestry base, commodity grain base, temperate biological species gene pool and ecological barrier in China [4,5]. The formation of ecological spatial pattern in Northeast China is closely related to its geological and geomorphological background. Mountain landforms often correspond to forest ecosystems, while plain areas mostly correspond to agricultural and grassland ecosystems.

Coupling degree is often used to describe the degree of interaction and influence between two or more systems. It determines the order and structure of the system when it reaches the critical region [6,7]. Synergism is a theory that explores the coordination effect amongst the elements of a multi–element system. This effect leads to the overall identity, structural stability, evolutionary order and functional optimisation of the system. Coupling degree evaluation and coordination effect analysis are combined to form a coupling coordination degree (CCD) model, which is used to evaluate the coordinated development degree of systems [8]. The model has a clear meaning and simple calculation. It has been widely used in various geoscience fields [7,9]. In geoscience fields, it is common to study the coupling coordination relationship related to ecology. To support the implementation of ecological restoration and control projects, many scholars studied the coupling coordination of soil–vegetation system in different types or areas [8,10,11]. The spatial–temporal pattern evolution characteristics of the eco–environment coupling coordination stage and quality level in the Hexi Corridor were evaluated in terms of environmental carrying capacity and ecological elasticity [12]. To provide a strong basis for regional ecological protection and social economic development, a theoretical framework for the evaluation of CCD was constructed and used to evaluate of coupling coordination characteristics of ecosystem, natural and human elements. Most of these studies were usually based on urbanisation and eco–environment [9,13–15], spatial pattern and topography of poor counties [16], social–economy and carbon emissions [17] and energy–economy–ecosystem [18] aspect. Others were based on eco–environment and tourism [19,20], social economic development [21,22], regional development intensity [23], regional high–quality development [24,25], ‘three living’ system from the perspective of ecological civilisation construction [26–28] and mimicry and real living environment [29]. Literature search has shown the abundant results of coupling coordination analysis in geosciences. However, there is still a lack of studies that have been conducted on the coupling coordination between geology–geomorphology and ecology.

Vegetation is an important component of earth ecosystems. Numerous studies have been conducted to study the relationship between vegetation and climate change [5,30]. However, there are many studies on the single relationship between vegetation and geology [31,32] or between vegetation and geomorphology [4,33], but few studies [1,3] on the relationship between vegetation and geology–geomorphology comprehensive system. In the past, scholars focused on the information extraction method, spatial distribution pattern, change response and vegetation and geology–geomorphology correlation. Many scholars used remote sensing methods to study the response history of river geomorphological changes and vegetation, the interaction mechanism of watershed hydrological processes and riparian vegetation [34–37]. Some scholars applied the ground monitoring method to explore the influence of soil water dynamics under different forest vegetation covers on slope stability [38]. Some scholars comparatively studied the effects of vegetation and bedrock types on rock weathering and soil formation rate in two temperate forest basins. Bedrock type may be the main factor causing the differences between the two factors [39]. On the basis of topography, remote sensing imaging and field investigation, some scholars investigated the relationship between the morphological changes in mountain landform and vegetation pattern [33], the regulatory effect of different vegetation communities in gully steep slope development area on the soil and water loss of gully bank reconstruction [40], the connectivity mechanism between vegetation and landform in a mountain river system [41], the differences of vegetation dynamics between ephemeral and perennial streams in mountainous headwater catchments and the effects of hydrology, geology, landform, climate and human activities on vegetation dynamics [42], and the spatial change and correlation between vegetation and geographical environment variables after major geological disasters, such as landslides [31]. On the basis of multidisciplinary theory and methods, scholars explored the impact and correlation of lithology, geomorphology, human activities, and ecological restoration projects on vegetation in karst areas in China [32,43]. Spectral and spatiotemporal information are combined using seasonal changes in rocks and vegetation to improve the extraction method of rocky desertification information [44]. Studies on the semi–arid and semi–humid areas in northern China have shown that ecological engineering has improved the vegetation coverage, yield and biomass [45,46].

In view of the large and medium scale stability and the local small-scale dynamic variability of spatial characteristics of geology and geomorphology, the spatial configuration pattern of ecology and geology–geomorphology should be relatively stable on the medium scale of a region like Northeast China. However, the coupling characteristics and coordinated development status between geology–geomorphology and ecology are not very clear. Therefore, in order to address the aforementioned research questions, the objectives of this study are defined as three aspects: Firstly, we establish the spatial clustering relationship between geology–geomorphological elements and ecological elements in Northeast China, and carry out the comprehensive geology–geomorphological and ecological divisions. Secondly, with these divisions and from the perspective of system theory and synergy theory, we build an index system and use the coupling coordination model to quantitatively evaluate the spatial coupling coordination differences of geology–geomorphology and ecology in Northeast China. Thirdly and lastly, we study the temporal variation trend and spatial difference of the Normalized Difference Vegetation Index (NDVI) in Northeast China and various divisions, and put forward ecological management suggestions. This study is helpful to understand the regional eco–geological background conditions correctly, and is of great significance to spatial planning of national land and maintaining national ecological security.

## Materials and methods

### Study area

**Fig 1:**
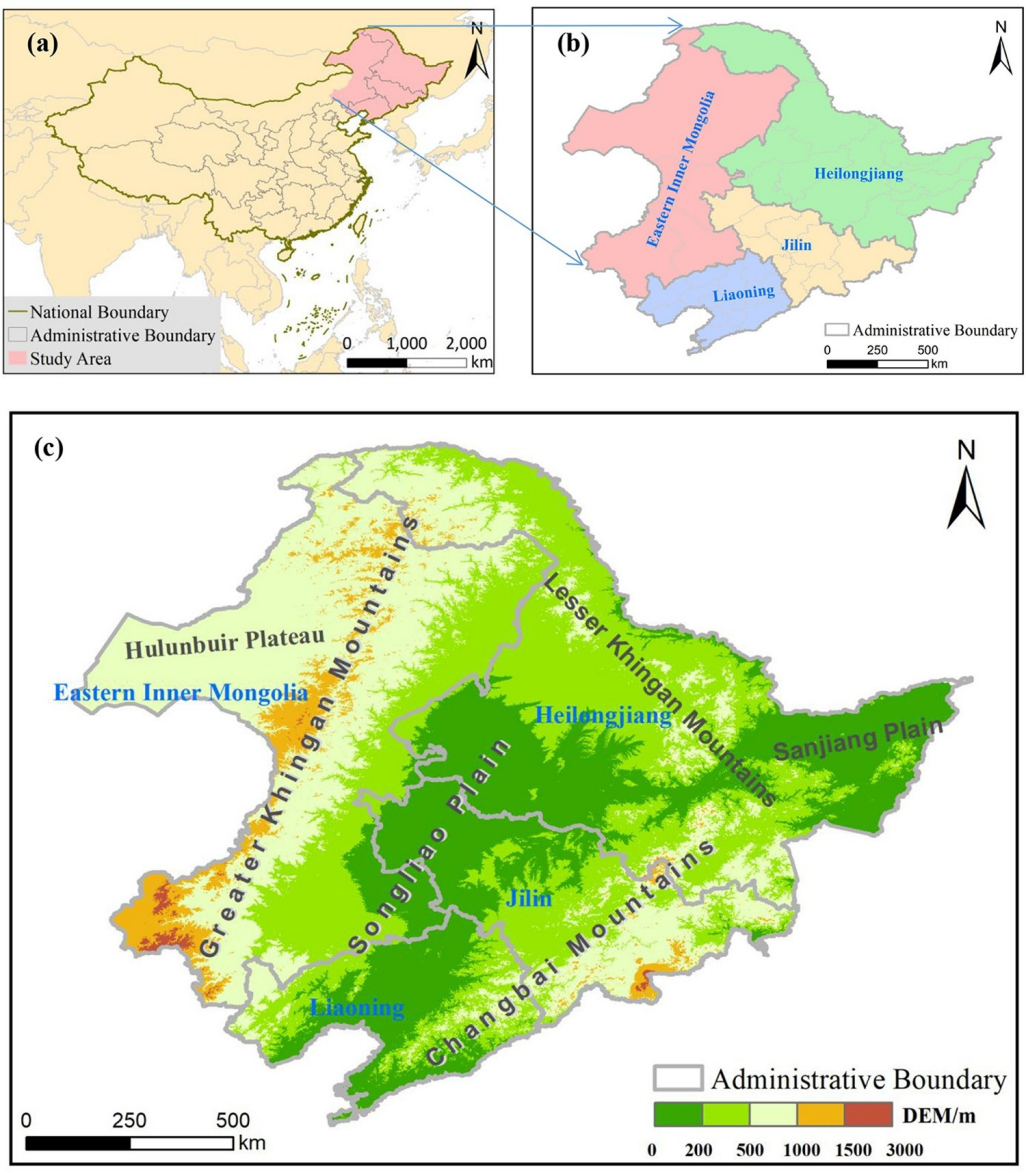
Location and geomorphological features of Northeast China. (a) Location; (b) Administrative region; (c) Geomorphological features. The original basemap was obtained from Natural Earth (http://www.naturalearthdata.com/) and was further processed using software ArcGIS 10.4 version. Digital elevation model was from the U.S. Geological Survey (https://www.usgs.gov/).

Northeast China is located between a longitude of 115°31ʹ and 135°05ʹ E and at a latitude of 38°43ʹ and 53°34ʹ N. It covers an area of approximately 1,240,000 km^2^, accounting for about 13% of China’s land area (Fig 1A). Its administrative region includes Heilongjiang Province, Jilin Province, Liaoning Province and eastern Inner Mongolia Autonomous Region (which comprises Hulunbuir City, Tongliao City, Chifeng City, and Xing’an League) (Fig 1B). It faces Korea across the Yalu River and Tumen River in the southeast, Russia in the east and north, Mongolia in the west, and the Bohai Sea and the Yellow Sea in the south [5,47].

Northeast China has a temperate monsoon climate. Its geological structure crosses two first–order tectonic units, namely, the North China block and Tianshan–Xingmeng orogenic system. The main geomorphological units include Greater Khingan Mountains, Lesser Khingan Mountains, Changbai Mountains, Hulunbuir Plateau, and Northeast Plain. The Northeast Plain is divided into Sanjiang Plain and Songliao Plain according to its spatial location (Fig 1C).

Northeast China is surrounded by mountains in the west, north and east and by plain in the south. The main ecosystem types are coniferous forest, mixed coniferous and broad–leaved forest, deciduous broad–leaved forest, meadow, grassland, grass, broad–leaved shrub, river, and swamp amongst others [47,48].

### Datasets

The following data are used: geological data from *1*:*1*,*000*,*000 Tectonic Map of China* (2009) and *1*:*500*,*000 Geological Map of China* (1999), landform type data from *1*:*1*,*000*,*000 Geomorphological Map of China* (2009), fault data from *1*:*4*,*000*,*000 Geomorphological Map of China* (2014) and crustal stability data from *the Seismic Parameter Zoning Map of China* (GB18306–2015) (2015).

For digital elevation model (DEM) data, the SRTM1 product (2000), which was jointly measured by NASA and NIMA, is selected, with a resolution of 30 m and WGS84 ellipsoid projection. Based on the DEM data, slope data are extracted by using ArcGIS 10.4. The river system data are obtained from *1*:*250*,*000 Geographical Elements Map of China* (2002). The topsoil organic carbon (TOC) and pH values are acquired from *Chinese Soil Dataset* based on the world soil database (*Harmonized World Soil Database*, HWSD) (v1.1) (2009).

Ecosystem data are from *the Terrestrial Ecosystem Database of China* (2010), which comes from *the Ecosystem Assessment and Ecological Security Database Platform of China* (http://www.ecosystem.csdb.cn). Vegetation types are from *1*:*1*,*000*,*000 Vegetation Type Map of China* (2001). *The Annual Normalized Difference Vegetation Index (NDVI) Spatial Distribution Dataset of China* (1998–2018) and *the Net Primary Productivity (NPP) data* (2000–2010) are from *the Resources and Environmental Science and Data Centre of Chinese Academy of Sciences* (https://www.resdc.cn). The MOD13A1 NDVI data of 500 m spatial resolution (2001–2020) are from *the LAADS DAAC data distribution system platform* of Earth Observation System Data and Information System (EOSDIS) of NASA (https://ladsweb.modaps.eosdis.nasa.gov). The human population density data are determined from the bulletins of the seventh national population census of Heilongjiang Province, Jilin Province, Liaoning Province and Inner Mongolia Autonomous Region (2020).

The main data source of remote sensing image is the three-dimensional (3D) land image of Northeast China, which was produced by the latest Landsat–8 OLI images of 15 m spatial resolution (2017–2018) and DEM of 30 m spatial resolution (2000). Landsat–8 images were provided by the website of the United States Geological Survey (USGS). After radiometric calibration, atmospheric correction, image fusion, image mosaic and clipping, Landsat–8 images are combined with DEM to make a 3D image. The spatial positioning accuracy error is no more than 1 pixel.

The data used in this study are processed on ENVI, ArcGIS, MATLAB, Microsoft Excel software platforms. All data are standardised and fed in two types. One is vector data, including geological data (lithology), fault, landform type, river system, ecosystem and vegetation type data. The other is grid data, including DEM, slope, crustal stability, TOC, pH, NDVI, NPP and the human population density data. The grid data are resampled into grids with 1km spatial resolution.

### Methods

#### Eco–geological division method

Computer automatic classification and visual interpretation are two main methods in image classification [49,50]. To improve the efficiency and reliability of data extraction, the human-computer interaction method combining computer automatic classification and visual interpretation is widely adopted in image classification of complex areas and comprehensive zoning research [51,52]. In this paper, the ecological and geology–geomorphological division is abbreviated as eco–geological division. It is a comprehensive zoning on the basis of the data of geology, geomorphology, ecosystem, vegetation types and 3D remote sensing image of Northeast China. The human–computer interaction method is used to complete the division process by basing on lithology, altitude, slope, main ecosystem types and vegetation distribution, considering the integrity of terrain contour and using spatial clustering and overlay analysis tools in ArcGIS 10.4. Eco–geological dividing is the first and critical step of this study. With the division results, we can move to the next step to explore the coupling coordination relationship between geology–geomorphology and ecology.

#### Construction of evaluation index system

According to the correlation of geology–geomorphology and ecosystem structure and function, 17 evaluation indices representing geology–geomorphology subsystem and ecology subsystem are selected. The index attributes are calibrated in positive and negative directions (Table 1). Positive indicators represent factors that promote the good development of the system (the higher the positive index value is, the better the coupling coordination of the system will be), and negative indicators interfere with these processes.

**Table 1:** Evaluation index system of CCD between geology–geomorphology and ecology.

Target layer	Criterion layer	Index layer	References	Indicator meaning	Index attribute
Complex system of geology–geomorphology and ecology	Geology–geomorphology subsystem	Geological complexity (A1)	Xie et al. (2018), Liao et al. (2002), Zhao et al. (1995) [53–55]	*P* = *n*/*S* *P* is the geological complexity, *n* is the individual number of different stratum and lithology, and *S* is the area of a region	-
		Mean altitude (A2)	Kong et al. (2017) [56]	The average value of altitude in a region	-
		Topographic relief (A3)	Zhou et al. (2017) [16,57]	The difference between the maximum and minimum elevation in a region	-
		Slope (A4)	Zhou et al. (2017) [16]	The average value of terrain slope of surface unit in a region	-
		River density (A5)	Zhou et al. (2017) [16]	The ratio of the total length of the river system to the area of a region	-
		Fracture density (A6)	Zheng et al.(2010) [57]	The ratio of the extension length of fault development to the area of a region	-
		Crustal stability (A7)	Zheng et al.(2010), Ma et al. (2021) [57,58]	The stability degree of a region on the surface of the earth’s crust under the action of the earth’s internal dynamics. It is expressed by the average value of the peak acceleration of regional ground motion in this study	-
		Topsoil organic carbon (A8)	Kong et al. (2017) [56]	Proportion of all carbonaceous organic matter in topsoil (0–30 cm)	+
		Topsoil pH (A9)	Li et al. (2019) [59]	Acidity and alkalinity of topsoil (0–30 cm)	-
	Ecology subsystem	Vegetation fraction (fc) (B1)	Zheng et al.(2010), Li et al. (2004) [57,60]	$fc=(NDVI-NDVI_{soil})/(NDVI_{veg}-NDVI_{soil})$	+
		Net primary productivity (NPP) of vegetation (B2)	Yang et al. (2020) [12]	The NPP data (2000–2010) are from *Resources and Environmental Science and Data Center of Chinese Academy of Sciences* (https://www.resdc.cn). It is calculated based on light energy utilization model GLO_PEM	+
		Habitat fragmentation of ecosystem (B3)	Ministry of Ecology and environment (MEE) of the People’s Republic of China (2020) [61]	*F*_*i*_ = *N*_*i*_/*S*_*i*_ *F*_*i*_ is the fragmentation of a certain type of ecosystem, *N*_*i*_ is the number of patches in a certain ecosystem, and *S*_*i*_ is the total area of a certain ecosystem	-
		Shannon–Wienner diversity index (B4)	Kong et al. (2017), H. TASIKEN et al. (2021) [56,62]	$H=-\sum \left[P_{i}*\ln(P_{i})\right]$ *H* is the Shannon–Wienner diversity index, *P*_*i*_ = *n*/*N*, *n* is the number of individuals of a vegetation type, and *N* is the total number of individuals of each vegetation type	+
		Margalef richness index (B5)	Kong et al. (2017), H. TASIKEN et al. (2021) [56,62]	*R* = (*S*−1)/ln(*N*) *R* is Margalef richness index, *S* is the total number of vegetation types, *N* is the total number of individuals of each vegetation type	+
		Pielou evenness index (B6)	Kong et al. (2017), H. TASIKEN et al. (2021) [56,62]	*E* = *H*’/ln(*S*) *E* is the Pielou evenness index, *H’* is Shannon–Wienner diversity index of vegetation types, *S* is the total number of vegetation types	+
		Simpson diversity index (B7)	H. TASIKEN et al. (2021) [62]	$D=1-\sum_{i=1}^{k}P_{i}^{2}$ *D* is the Simpson diversity index, *k* is the number of vegetation types, the proportion of the individual number of vegetation type *i* to the total number of individuals in the region is *p*_*i*_, and the joint probability of two individuals randomly selected from species *i* is $P_{i}^{2}$	+
		Human population density (B8)	Zhou et al. (2017) [16]	Human population per unit area, indicates the human population density of a region	-

17 indices are standardised. For *n* divisions and *m* indices, *x*_*ij*_ and $x_{ij}^{'}$ represent the original and the standardised values of the j–th index of the i–th division, respectively, and *i =* 1, 2, …, *n*; *j =* 1, 2, …, *m*. In this study, *n* = 9, and *m =* 17 [18]. The standardised formulas of positive and negative indicators are shown in Eqs (1) and (2), respectively.


$$x_{ij}^{'}=\frac{x_{ij}-\min\{x_{1j},\cdots ,x_{nj}\}}{\max\{x_{1j},\cdots ,x_{nj}\}-\min\{x_{1j},\cdots ,x_{nj}\}}$$ (1)

$$x_{ij}^{'}=\frac{\max\{x_{1j},\cdots ,x_{nj}\}-x_{ij}}{\max\{x_{1j},\cdots ,x_{nj}\}-\min\{x_{1j},\cdots ,x_{nj}\}}$$ (2)

#### Index weight assignment method

In accordance with previous studies [15,63,64], in this research, analytic hierarchy process (AHP) is used for subjective weighting, entropy weighting method is utilised for objective weighting, and game theory comprehensive weighting method is applied to calculate the comprehensive weight of each index. This index weighting method can give consideration to the preference of decision–makers for attributes and reduce the subjective arbitrariness of weighting to achieve the unity of subjective and objective weighting.

*AHP*. AHP is a systematic analysis method, which can make complex problems hierarchical and qualitative problems quantitative [65]. It is one of the most commonly used subjective weighting methods. The basic steps include the following: Firstly, a hierarchical structure model is built. Then, a judgement matrix is constructed, and relevant experts are invited to score according to the relative importance of two factors. Finally, the weight of each factor and the maximum eigenvalue are calculated, and consistency is tested. This study requires the consistency ratio less than 0.1 to ensure that the weight determined is effective. The specific algorithms can be referred to reference [66].

*Entropy weight*. Entropy weight method is a common objective weighting method, which depends on the discreteness of data itself [18]. The dispersion degree of an index can be determined by calculating entropy. The higher the entropy value is, the greater the impact of the index on the comprehensive evaluation and the greater the weight will be. The algorithms can be referred to reference [15] and S1 File.

*Game theory*. Game theory comprehensive weighting method considers the advantages of both subjective and objective weighting. Firstly, the basic weight vector set is constructed according to AHP and entropy weight. Then, combination vectors are optimised and the optimal first derivative condition is obtained. Finally, the linear coefficient is obtained through normalisation, and the final comprehensive weights are calculated. The basic calculation method of game theory can be referred to reference [63] and S1 File.

#### Coupling coordination degree model

A coupling coordination degree (CCD) model is constructed to evaluate the spatial coupling coordination differences of geology–geomorphology and ecology in Northeast China. The model algorithm is shown in Eqs (3) to (7) [15].

$$C=2{\left\{\frac{G(x)\times E(y)}{{\left[G(x)+E(y)\right]}^{2}}\right\}}^{1/2}$$ (3)

$$G(x)=\sum_{i=1}^{m}ax_{i}$$ (4)

$$E(y)=\sum_{j=1}^{n}{\mathrm{by}}_{j}$$ (5)

$$D=\sqrt{C\cdot T}$$ (6)

T=α⋅G(x)+β⋅E(y) (7)

where *C* is the coupling degree of geology–geomorphology and ecology, and 0 ≤ *C* ≤ 1. The larger the value of *C* is, the stronger the coupling between the two subsystems will be. *G(x)* and *E(y)* are the comprehensive evaluation functions of geology–geomorphology and ecology, respectively. *x*_*i*_ and *y*_*i*_ are the normalised values of the indices of the geology–geomorphology and ecology subsystems, *a* and *b* are the weights of the indices, and *m* and *n* are the numbers of indices of the two subsystems. *D* is the coupling coordination degree, and 0 ≤ *D* ≤ 1. The larger the value of *D* is, the higher the overall level of the elements or system and the more coordination the development of the two subsystems will be. *T* is the comprehensive coordination index, which reflects the contribution of the subsystems or elements to the coupling coordination degree. *α* and *β* are the weights of each subsystem, considering the equal importance of geology–geomorphology and ecology, where *α* = *β* = 0.5. *E(y)/G(x)* represents the relative development degree of the comparison between the two subsystems. The types and characteristics of *C*, *D* and *E(y)/G(x)* are shown in Table 2.

**Table 2:** Types and characteristics of *C*, *D* and *E(y)/G(x)* in the research.

Parameter	Meaning	Types and characteristics
*C*	Coupling degree	*C* = 0, disorderly development stage; 0 < *C* ≤ 0.3, very low coupling; 0.3 < *C* ≤ 0.5, antagonism period; 0.5 < *C* ≤ 0.8, running in period; 0.8 < *C* ≤ 1, very high coupling, the system has entered an orderly development stage.
*D*	Coupling coordination degree	0 ≤ *D* ≤ 0.4, serious imbalance; 0.4 < *D* ≤ 0.5, moderate imbalance; 0.5 < *D* ≤ 0.6, mild imbalance; 0.6 < *D* ≤ 0.7, low coordination; 0.7 < *D* ≤ 0.8, moderate coordination; 0.8 < *D* ≤ 1, high coordination.
*E(y)/G(x)*	Relative development degree of subsystems	*E(y)/G(x)* < 0.8, ecology lag type; 0.8 ≤ *E(y)/G(x)* ≤ 1.2, commonly lag type when 0 ≤ *D* ≤ 0.6, simultaneous development type when 0.6 < *D* ≤ 1; *E(y)/G(x)* > 1.2, geology–geomorphology lag type.

#### Sen+Mann–Kendall method

The variation period of geological and geomorphological characteristics is long, so the research on the coupling coordination between ecology and geology–geomorphology in this study is limited to the spatial dimension. In order to supplement the deficiency of temporal dimension, based on the MOD13A1 NDVI data from 2001 to 2020, the annual NDVI values are calculated by the maximum value synthesis method, and applied to analysing the variation trend and significant test of Sen+Mann–Kendall (Sen+MK). The advantage of Sen+MK method is that it can eliminate the influence of outliers in long–time series data analysis [67]. Sen’s slope is calculated as Eq (8), and the MK method is defined according to Eqs (9) to (11) [68].


$$\beta =Median\left(\frac{x_{j}-x_{i}}{j-i}\right),j>i$$ (8)

where *β* represents the variation trend, *x*_*j*_ and *x*_*i*_ are the sequence values at time *j* and *i*, respectively, and *Median*() is the median function. *β* > 0 indicates that the temporal series shows an increasing trend, *β* < 0 indicates that the temporal series shows a decreasing trend.


$$Z=\left\{ \begin{array}{ll} \frac{S}{\sqrt{Var(S)}} & \left(S>0\right)\\ 0 & \left(S=0\right)\\ \frac{S+1}{\sqrt{Var(S)}} & \left(S<0\right) \end{array}\right.$$ (9)

$$S=\sum_{i=1}^{n-1}\sum_{j=i+1}^{n}sign(x_{j}-x_{i})$$ (10)

$$sign(x_{j}-x_{i})=\left\{ \begin{array}{ll} 1 & \left(x_{j}-x_{i}>0\right)\\ 0 & \left(x_{j}-x_{i}=0\right)\\ -1 & \left(x_{j}-x_{i}<0\right) \end{array}\right.$$ (11)

where *Z* is the statistical value of the standardised test, *S* is the statistical value of the test, *Var* (*S*) is the variance, *sign* () is the sign function, *x*_*j*_ and *x*_*i*_ are the sequence data at time *j* and *i*, *n* is the number of data, for all *i*, *j* ≤ n, and *i* ≠ *j*. When *n* ≥ 8, *S* is approximately normal distribution, and its mean value is $E(S)=0,\;Var(S)=\frac{n(n-1)(2n+5)}{18}$. When Z > 0, the temporal series data show an increasing trend; when Z < 0, the temporal series data show a decreasing trend. At a given significant level α, if $|Z|>Z_{1-\frac{\alpha }{2}}$, indicating that the hypothesis there is no trend is rejected, there is an obvious variation trend in the temporal series data. When |Z| is greater than 1.96, it means that it has passed the significant test with 95% confidence (i.e. α = 0.05).

#### Methods implementation and data statistics

Many software and computer programs are used to implement these methods. Among them, ENVI 5.3 software is used to process remote sensing images. YAAHP software is used for AHP. Batch codes in Python program and MATLAB R2018b software are adopted to process NDVI time series data and calculate Sen+MK variation trend. Microsoft Excel is applied for calculating values of entropy weight, game theory and CCD and statistical analysis and charts. ArcGIS 10.4 software is utilised for data processing, eco–geological division, spatial analysis of CCD and Sen+MK and mapping. The Sen+MK analysis results are exported from ArcGIS 10.4 to Microsoft Excel for pixel statistics. The number and percentage of pixels with significant increasing and decreasing trends not only in the whole Northeast China, but also in each division are counted.

## Results

### Nine eco–geological divisions

**Fig 2:**
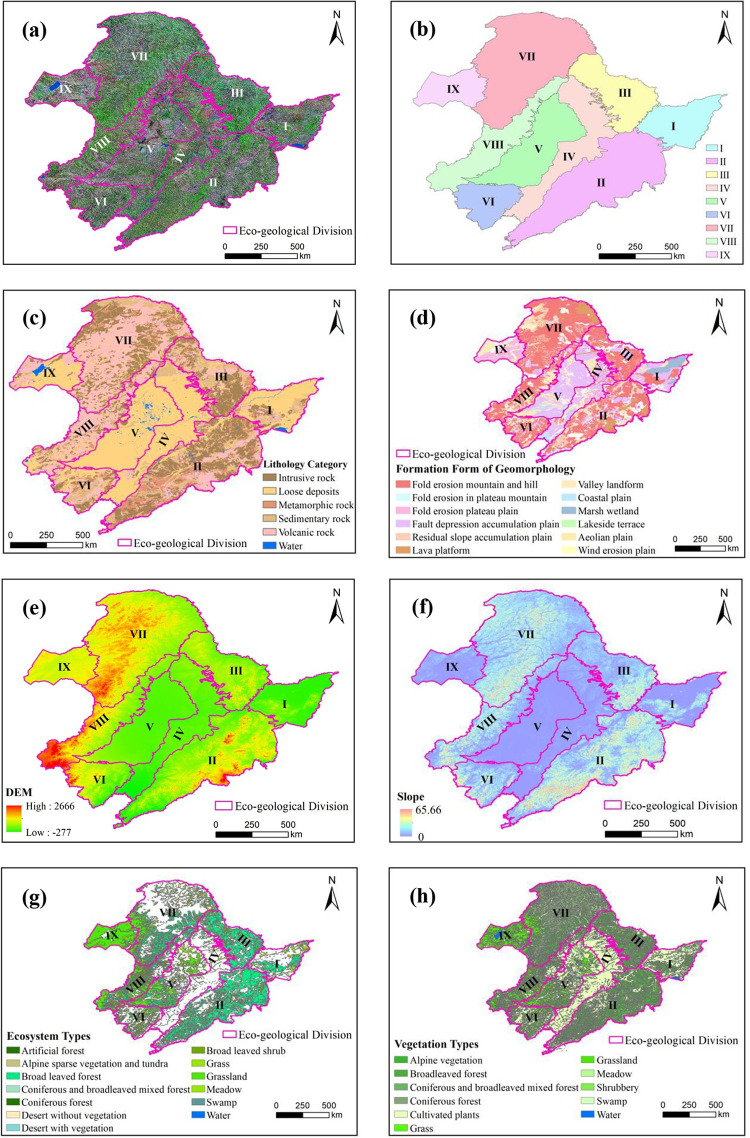
Eco–geological division of Northeast China. (a) Remote sensing image and divisions; (b) Nine eco–geological divisions; (c) Lithology and divisions; (d) Landform and divisions; (e) Altitude and divisions; (f) Slope and divisions; (g) Ecosystem and divisions; (h) Vegetation type and divisions. Digital elevation model and Landsat–8 images were provided by USGS EROS (Earth Resources Observatory and Science (EROS) Center) (http://eros.usgs.gov/#). All maps were further processed using ArcGIS 10.4 software.

Northeast China is divided into nine eco–geological divisions, which are: (Ⅰ) Sanjiang Plain, (Ⅱ) the Changbai Mountains, (Ⅲ) the Lesser Khingan Mountains (LKM), (Ⅳ) eastern Songliao Plain, (Ⅴ) central and western Songliao Plain, (Ⅵ) upper reaches of the West Liao River and the western Liaoning Mountains, abbreviated as ‘western Liaoning Mountains’, (Ⅶ) the central and northern Greater Khingan Mountains (GKM), (Ⅷ) southern GKM, and (Ⅸ) Hulunbuir Plateau. The division results are shown in Fig 2, and eco–geological characteristics of each division are referred to S1 Table.

### Standardised value and weighting of index system

**Fig 3:**
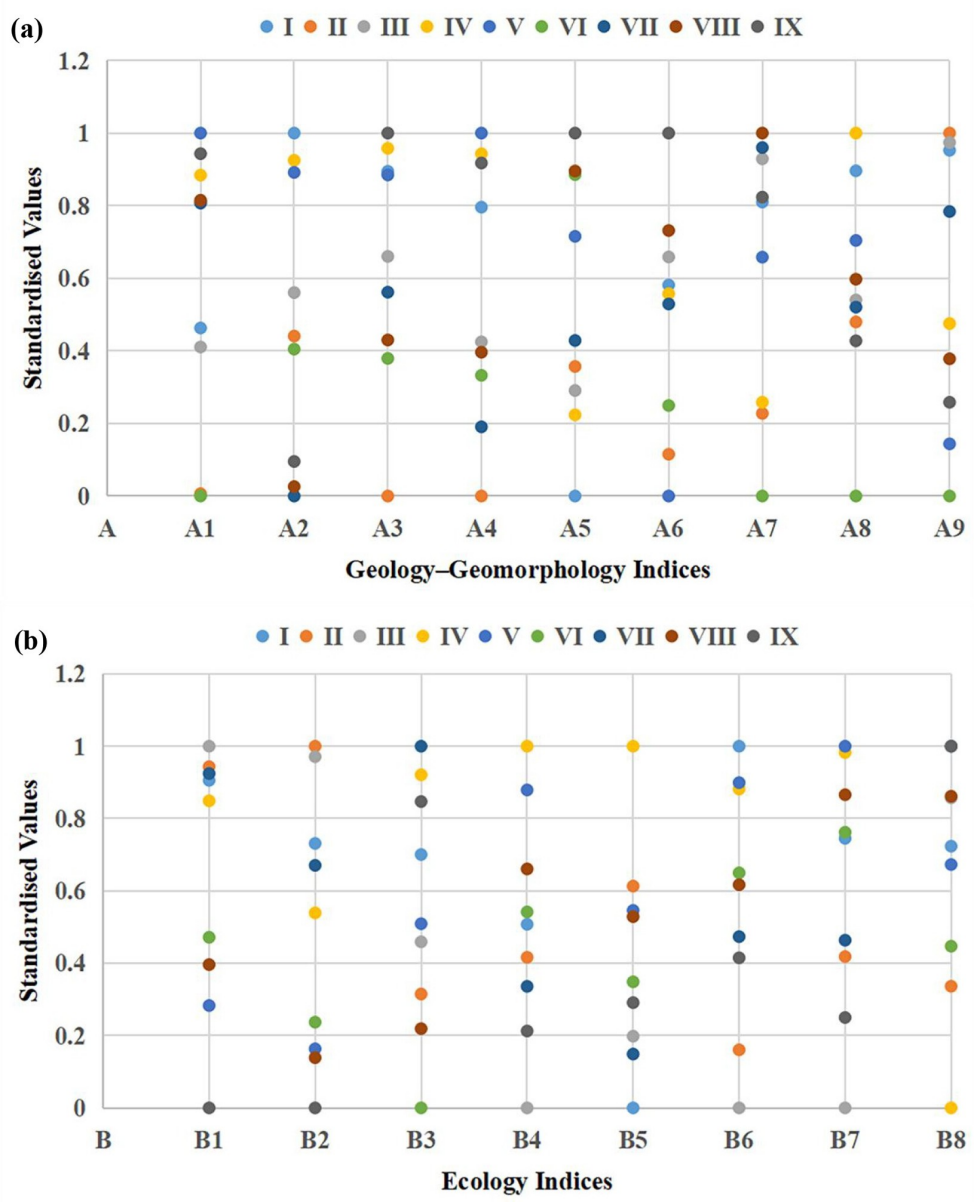
Standardised values of evaluation indices. (a) Geology–geomorphology indices; (b) Ecology indices.

A total of 17 evaluation indices representing geology–geomorphology and ecology are normalised to obtain the standardised index values (Fig 3). The linear coefficient $\beta_{k}^{*}$ is obtained through the normalisation of game theory method. The calculation results of the linear coefficient of geology–geomorphology subsystem are as follows: $\beta_{1}^{*}=0.9076$, and $\beta_{2}^{*}=0.0924$. The calculation results of the linear coefficient of the ecological subsystem are as follows: $\beta_{1}^{*}=0.9377$, and $\beta_{2}^{*}=0.0623$. The weights calculated by AHP, entropy weight and game theory are shown in Table 3.

**Table 3:** Results of index system weighting of geology–geomorphology and ecology in Northeast China.

Target layer	Criterion layer	Index layer	Index weight		
			AHP	Entropy weight	Game theory
Complex system of geology–geomorphology and ecology	Geology–geomorphology subsystem	Geological complexity (A1)	0.23	0.13	0.22
		Mean altitude (A2)	0.09	0.17	0.10
		Topographic relief (A3)	0.14	0.08	0.14
		Slope (A4)	0.14	0.11	0.14
		River density (A5)	0.05	0.11	0.06
		Fracture density (A6)	0.02	0.11	0.03
		Crustal stability (A7)	0.02	0.10	0.03
		Topsoil organic carbon (A8)	0.09	0.07	0.09
		Topsoil pH (A9)	0.21	0.12	0.20
	Ecology subsystem	Vegetation fraction (fc) (B1)	0.22	0.11	0.22
		Net primary productivity (NPP) of vegetation (B2)	0.23	0.17	0.23
		Habitat fragmentation of ecosystem (B3)	0.12	0.12	0.12
		Shannon–Wienner diversity index (B4)	0.11	0.12	0.11
		Margalef richness index (B5)	0.07	0.15	0.07
		Pielou evenness index (B6)	0.03	0.12	0.03
		Simpson diversity index (B7)	0.07	0.11	0.07
		Human population density (B8)	0.16	0.10	0.15

In the subsystem of geology–geomorphology, the order of index weight from large to small is as follows: geological complexity > topsoil pH > topographic relief = slope > mean altitude > topsoil organic carbon > river density > fracture density = crustal stability. In the subsystem of ecology, the order of index weight from large to small is as follows: NPP > vegetation fraction > human population density > habitat fragmentation > Sannon–Wiener diversity index > Margalef richness index = Simpson diversity index > Pielou evenness index.

### Calculation and analysis of CCD

**Fig 4:**
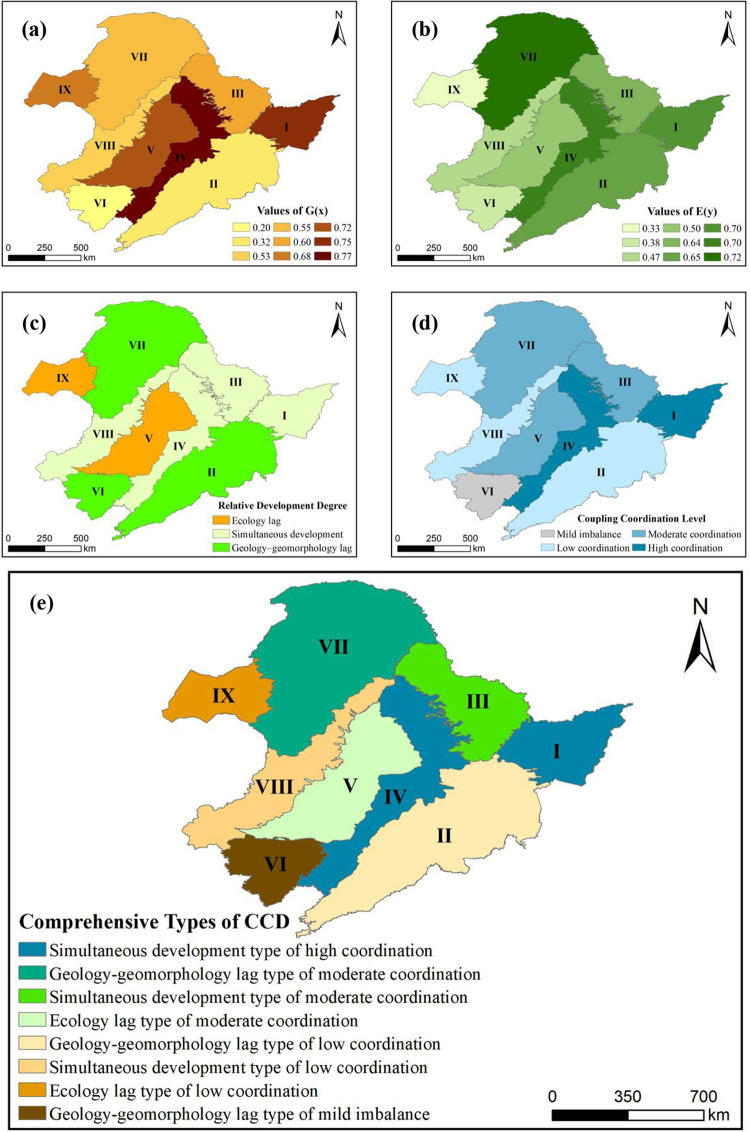
Spatial differentiation of coupling coordination between geology–geomorphology and ecology in Northeast China. (a) Comprehensive evaluation results of geology–geomorphology; (b) Comprehensive evaluation results of ecology; (c) Relative development degree between geology–geomorphology and ecology; (d) Coupling coordination degree between geology–geomorphology and ecology; (e) Comprehensive evaluation of development level and coupling coordination type. All maps were processed using software ArcGIS 10.4 version.

Through the CCD model, the value and the ratio of two subsystems comprehensive evaluation functions, the coupling degree and the coupling coordination degree of each division are obtained. The results are shown in Table 4. C stands for coupling stage, D represents coupling coordination level and E(y)/G(x) represents relative development level. The spatial differentiation results of comprehensive evaluation of two subsystems, relative development level and coupling coordination degree types of nine divisions are shown in Fig 4.

**Table 4:** CCD evaluation results of different eco–geological divisions in Northeast China.

Division No.	G(x)	E(y)	C	Coupling stage	D	Coupling coordination level	E(y)/G(x)	Relative development level
**Ⅰ**	0.75	0.70	0.9994	Very high coupling	0.85	High coordination	0.93	Simultaneous development type
**Ⅱ**	0.32	0.65	0.9411	Very high coupling	0.67	Low coordination	2.02	Geology–geomorphology lag type
**Ⅲ**	0.60	0.64	0.9996	Very high coupling	0.79	Moderate coordination	1.06	Simultaneous development type
**Ⅳ**	0.77	0.70	0.9987	Very high coupling	0.86	High coordination	0.90	Simultaneous development type
**Ⅴ**	0.72	0.50	0.9830	Very high coupling	0.77	Moderate coordination	0.69	Ecology lag type
**Ⅵ**	0.20	0.38	0.9460	Very high coupling	0.52	Mild imbalance	1.96	Geology–geomorphology lag type
**Ⅶ**	0.55	0.72	0.9914	Very high coupling	0.79	Moderate coordination	1.30	Geology–geomorphology lag type
**Ⅷ**	0.53	0.47	0.9983	Very high coupling	0.70	Low coordination	0.89	Simultaneous development type
**Ⅸ**	0.68	0.33	0.9364	Very high coupling	0.69	Low coordination	0.48	Ecology lag type

The calculation results show that:

According to *G(x)*, the values of Ⅳ, Ⅰ and Ⅴ are larger, which are all above 0.7; the values of Ⅸ, Ⅲ, Ⅶ and Ⅷ are in the middle, ranging from 0.5 to 0.7; and the values of Ⅱ and Ⅵ are small, which are below 0.4, i.e. Ⅵ is only 0.20.

According to *E(y)*, Ⅶ has the largest value amongst the nine divisions, which is 0.72. Its value is followed by Ⅳ, Ⅰ, Ⅱ and Ⅲ, which are between 0.6 and 0.7; the values of Ⅴ, Ⅷ, Ⅵ and Ⅸ are small, ranging from 0.3 to 0.5.

At the coupling stage, the value of *C* (coupling degree) in the nine divisions is more than 0.9, which is at a high coupling stage. This result indicates that the interaction between geology–geomorphology and ecology in Northeast China is strong and highly correlated in long–term historical evolution.

For the coupling coordination type, the value of *D* (coupling coordination degree) in nine divisions is more than 0.5. This result indicates that the coupling coordination level of geology–geomorphology and ecology in Northeast China is good. There are two high–level, three medium–level and three low–level coordination types and one mild imbalance type. Amongst them, Ⅵ belongs to mild imbalance, with the value of *D* between 0.5 and 0.6; Ⅱ, Ⅸ and Ⅷ, belong to low coordination, with the values between 0.6 and 0.7; Ⅴ, Ⅲ and Ⅶ have values between 0.7 and 0.8, which belong to moderate coordination; Ⅰ and Ⅳ belong to high coordination, which is between 0.8 and 1.

For the relative development level of subsystems, Ⅱ, Ⅵ and Ⅶ belong to the types of geology–geomorphology lag, Ⅴ and Ⅸ belong to the ecology lag types, and Ⅰ, Ⅲ, Ⅳ and Ⅷ belong to the simultaneous development types of ecology and geology–geomorphology.

For the comprehensive evaluation results,

- The two divisions of high coordination, namely, Ⅰ and Ⅳ, are plain divisions where the typical black soil belt is located. The ecology and geology–geomorphology of the two divisions develop simultaneously, and the comprehensive evaluation function values of the subsystems are higher than or equal to 0.70.
- Among the three divisions of moderate coordination, there are not only simultaneous type but also ecology lag type and geology–geomorphology lag type. Among them, mountain division Ⅲ belongs to the simultaneous type, plain division V belongs to the ecology lag type and mountain division Ⅶ belongs to the geology–geomorphology lag type.
- In the three divisions of low coordination, one of the comprehensive evaluation function values of the two subsystems is lower than 0.5, and geology–geomorphology lag, ecology lag and simultaneous development type coexist. Mountain divisions Ⅱ and Ⅷ belong to the geology–geomorphology lag type and the simultaneous type, respectively, and plateau division Ⅸ belongs to the ecology lag type.
- In the division of mild imbalance, the comprehensive evaluation function values of the two subsystems are lower than 0.4. Mountain division Ⅵ belongs to the geology–geomorphology lag type of mild imbalance.

### Analysis of NDVI variation trend

**Fig 5:**
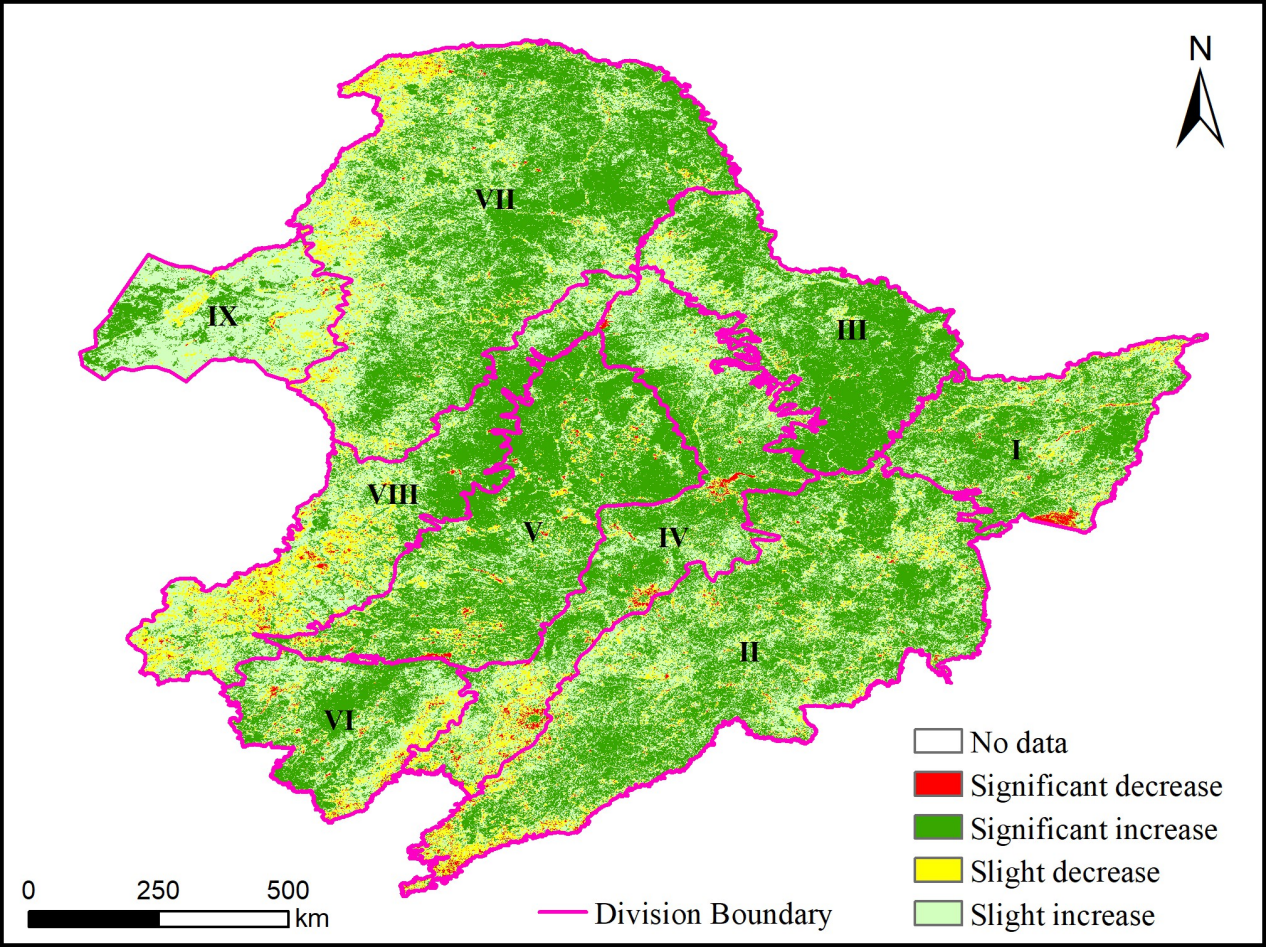
Results of NDVI trend analysis and significant test in Northeast China from 2001 to 2020. The NDVI data were obatined from NASA Earth Observatory (http://earthobservatory.nasa.gov/), and were further processed using Python program, MATLAB R2018b, ENVI 5.3 and ArcGIS 10.4 software.

The results of Sen’s slope calculation show that the variation trend of NDVI in Northeast China from 2001 to 2020 is between -0.054 and 0.055, NDVI generally shows an increasing trend, and only some areas in divisions Ⅳ, Ⅵ, Ⅶ and Ⅷ show a decreasing trend. The results of the MK significant test with α = 0.05 show that the areas with 95% confidence in Northeast China are mainly the areas with an increasing trend, which are concentrated in divisions Ⅶ, Ⅲ, Ⅴ and Ⅱ. The geographical areas are the central and northern GKM, the LKM, the central and western Songliao Plain and the Changbai Mountains. Most of the areas showing a decreasing trend did not pass the 0.05 test. However, the areas with a decreasing trend of 95% confidence were mainly distributed in divisions Ⅳ, Ⅷ and Ⅵ, especially the provincial capital cities and the urban agglomeration in central and southern Liaoning Province. According to whether the variation trend passes the significant test, the NDVI in Northeast China is divided into four categories: significant decrease, significant increase, slight decrease and slight increase (Fig 5).

**Fig 6:**
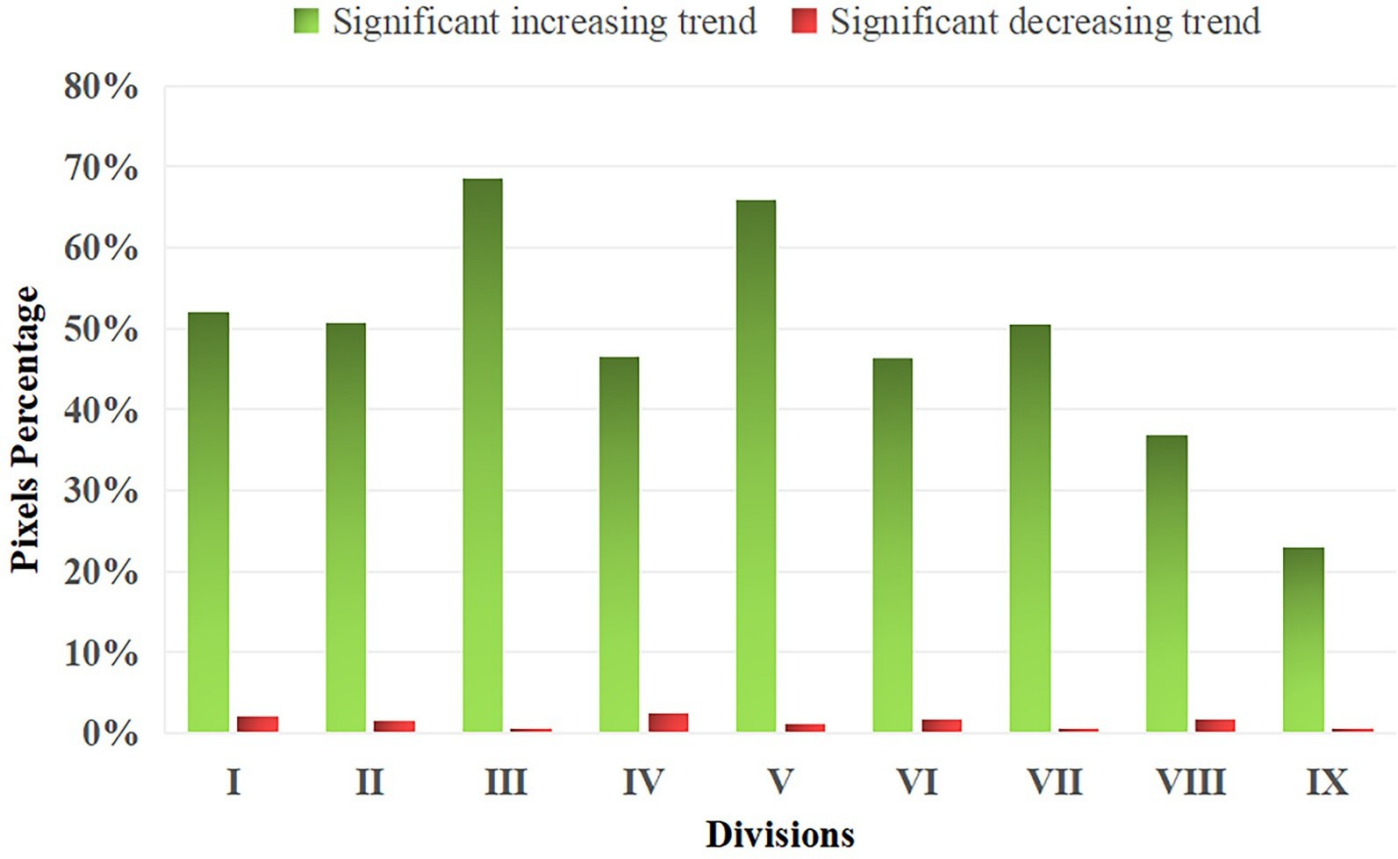
Percentage of significant variation trend of NDVI in the nine divisions in Northeast China.

The pixel statistical results show the significant variations of NDVI in the whole region and each division. In the whole region, 48.15% of the pixels that the NDVI variation trend failed 0.05 significant test and 51.85% of the pixels that passed the significant test. Among the pixels that passed the significance test, the pixels showing a significant decreasing trend account for 1.27%, the pixels showing a significant increasing trend account for 50.58%, and the proportion of unchanged pixels is close to 0, which can be ignored (Table 5). The percentage of pixels with a significant increasing trend of NDVI is quite different in each division, with the highest of 68.62% and the lowest of 23.02%. The order from high to low is Ⅲ>Ⅴ>Ⅰ>Ⅱ>Ⅶ>Ⅳ>Ⅵ>Ⅷ>Ⅸ. The percentage of pixels with a significant decreasing trend in each division is very small, and the difference is not obvious; the highest is 2.55%, the lowest is 0.23%, and the order is Ⅳ>Ⅰ>Ⅵ>Ⅷ>Ⅱ>Ⅴ>Ⅸ>Ⅶ>Ⅲ (Table 5 and Fig 6).

**Table 5:** Statistical results of NDVI variations trend Pixels of Northeast China.

Division NO.	Pixels	Pixels that fail the 0.05 test		Pixels that pass the 0.05 test					
				Significant decrease		Significant increase		Sen’s slope with confidence 95%	
	Sum	Count	Percentage	Count	Percentage	Count	Percentage	Max	Min
**Ⅰ**	298359	137074	45.94%	6025	2.02%	155260	52.04%	0.031	-0.040
**Ⅱ**	808470	386785	47.84%	11699	1.45%	409963	50.71%	0.039	-0.039
**Ⅲ**	369589	115120	31.15%	867	0.23%	253602	68.62%	0.028	-0.033
**Ⅳ**	459654	234357	50.99%	11710	2.55%	213581	46.47%	0.044	-0.053
**Ⅴ**	479785	158036	32.94%	5496	1.15%	316253	65.92%	0.046	-0.054
**Ⅵ**	225380	116744	51.80%	3988	1.77%	104645	46.43%	0.055	-0.042
**Ⅶ**	929621	455019	48.95%	4996	0.54%	469606	50.52%	0.029	-0.034
**Ⅷ**	402575	247468	61.47%	7088	1.76%	148019	36.77%	0.027	-0.043
**Ⅸ**	219672	167875	76.42%	1219	0.55%	50578	23.02%	0.046	-0.031
**Whole region**	4195834	2020305	48.15%	53185	1.27%	2122311	50.58%	0.055	-0.054

## Discussion

### Optimisation of index weighting method

This study improved the weight assignment method, introduced the comprehensive weighting method of game theory to optimise the index weight, balanced the shortcomings of the subjective weighting method and objective weighting method. In the past, scholars mostly used a single method to calculate the index weight of coupling coordination degree analysis [13,18,66], such as subjective AHP, with full consideration of expert experience. They also likely applied the objective entropy weight method based entirely on the mathematical properties and relations of the data themselves. Thus, this study presents the unity of subjective and objective methods.

### Reliability of coupling coordination evaluation

The CCD model has clear significance and simple calculation. Its application in many fields of geoscience has shown the reliability of the model. Due to the long change period of geological and geomorphological characteristics, this study only carries out the spatial coupling coordination feature analysis of ecology and geology–geomorphology by using multi–year average value or basic background data. Climate change is not considered in the evaluation process, based on the characteristics of climate sensitivity and relative stability of geology–geomorphology. We want to find out the spatial differences of regional eco–geological background conditions, simplify the complexity of earth system research, and pave the way for future research on the relationship between climate change and ecosystem.

Eco–geological dividing is a comprehensive evaluation and regional division method of the spatial cluster characteristics of geology–geomorphology and ecology. This research method is convenient to visually show the spatial cluster characteristics and type differences of ecology and geology–geomorphology in Northeast China on the map, that is, what kind of ecosystem is usually interdependent with what geological and geomorphological conditions. However, this method cannot describe interaction characteristics and coordination development status of geology–geomorphology and ecology. The CCD model solves the problem of quantitatively evaluating the coupling coordination relationship between the two subsystems. Through the evaluation, the advantages, problems and the degree of coupling coordination in geology–geomorphology and ecology of each division are shown. The evaluation of CCD model is a deeper evaluation and analysis after eco–geological dividing. The results of CCD model are consistent with the previous research conclusions on the geology–geomorphological conditions and ecosystems in Northeast China [48,69,70]. As an important commodity grain base, the plain landform, Quaternary sediments, and black soil resources in Northeast China are often closely related to agricultural ecosystem and grassland ecosystems, which breed high–quality vegetation NPP, such as the Sanjiang Plain and the Songliao Plain. We classify grasslands as natural ecosystems here, because the local government has adopted a strict protection policy to protect grassland from human activities since 2000 in Northeast China [71]. As an ecological barrier in China, mountains and hilly landforms and complex geological conditions in Northeast China are often closely related to the forest ecosystems, such as the LKM, the GKM and the Changbai Mountains. However, the fragile geology–geomorphological or ecological conditions will lead to the low eco–geological coupling coordination, such as the Changbai Mountains, the Hulunbuir Plateau and the southern GKM. Sometimes they even lead to mild imbalance, such as the western Liaoning Mountainous. It can be seen that the combination of eco–geological dividing and CCD model evaluation is a practical way to quantitatively evaluate the coupling coordination relationship between geology–geomorphology and ecology.

From the perspective of the eco–geological coupling coordination level, Division Ⅶ (north and center of the GKM, *D* = 0.79) and Division Ⅱ (the Changbai Mountains, *D* = 0.67) belong to moderate and low coordination types. Division I (Sanjiang Plain, *D* = 0.85) and Division Ⅳ (eastern Songliao Plain, *D* = 0.86) belong to the high coordination type. This finding seems to be inconsistent with the conventional cognition of the good ecological environment in the Changbai Mountains and the GKM. The reasons are as follows: ① The coupling coordination level represents not only the condition of a subsystem but also the synergy level of ecology and geology–geomorphology. ② Soil is the key part of the interaction between geology–geomorphology and ecosystem. Sanjiang Plain and eastern Songliao Plain have a plain and platform terrain with superior geological and geomorphological conditions. Under the joint action of multiple factors on the earth’s surface, the ‘panda in the soil’, black soil resources have been developed. The soil is thick and fertile, with strong artificial transformation and utilisation. Agricultural utilisation, field management and man–made engineering measures have made the ecosystem exhibit obvious productivity advantages. ③ The GKM and the Changbai Mountains have superior ecological conditions, but their geological and geomorphological conditions are complex. In particular, the Changbai Mountains have strong hydraulic erosion and dense development of gullies and steep slopes. In comparison with ecological conditions, the two regions show geology–geomorphology lag, and the research results are consistent with the conventional cognition. ④ The value of D also indicates that the eco–geological coupling coordination level in north and center of the GKM is close to the high coordination type. The above points fully show that the evaluation results of the coupling coordination level are credible.

### Significance of NDVI trend analysis

Songliao Plain and Sanjiang Plain, located in the black land of Northeast China, have superior soil quality, which has laid a unique geographical advantage for their ecosystem productivity [72]. In recent years, the typical black land in Northeast China has had some problems, such as ecological land fragmentation and black soil degradation, because of urbanisation and unreasonable human disturbance [73], which is consistent with the significant decreasing trend of NDVI in divisions Ⅳ and Ⅵ in this study. However, due to the time lag of vegetation response to habitat change, the ecosystem degradation effect is not obvious in this study because of the adoption of the multi–year average value of ecology characteristic elements as the background data. In addition, it has been considered that active human intervention, land management and engineering measures have significantly slowed down the trend of ecological land fragmentation and black soil degradation at a regional scale [73]. This is also illustrated by the significant increasing trend of NDVI in divisions Ⅲ and Ⅴ in this study.

NDVI is the main expression factor of ecosystem structure and function. The NDVI variation trend analysis considers the possible impact of the ecosystem change in temporal series, which complements the lack of research on the spatial coupling coordination characteristics of ecology and geology–geomorphology. The percentage of pixels with a significant increasing trend of NDVI in divisions Ⅲ and Ⅴ exceeds 60%, indicating that the ecological conditions in the central and western Songliao Plain and the LKM are developing well. The Sanjiang Plain, Changbai Mountains and the GKM corresponding to divisions I, II and VII also have a significant increasing trend. Among these divisions, Ⅴ belongs to the ecological lag type, and the coupling coordination between ecology and geology–geomorphology may be improved with the improvement of ecology in the future. Similarly, the coupling coordination level and type of other divisions will change with time. The percentage of pixels with a significant increasing trend of NDVI in divisions Ⅳ, Ⅵ, Ⅷ and Ⅸ is less than 50%. The D values of divisions VI, VIII and IX are low, which are in mild imbalance or low coordination types. Although Division Ⅳ is high coupling coordination, there are many cities and human activities in the region. Ecological management and ecological construction should be strengthened in these divisions to promote the coupling coordination development of regional earth system elements.

## Conclusion

This study follows the principle of earth system science and divides nine eco–geological divisions. With the nine divisions, the CCD model is used to quantitatively evaluate the coupling coordination characteristics between geology–geomorphology and ecology in Northeast China. The results show that the coupling effect between geology–geomorphology and ecology is strong, and the CCD is above 0.5. The coupling coordination level of geology–geomorphology and ecology in the nine divisions is high as a whole, but the spatial differentiation is obvious. There are two high–level, three medium–level and three low–level coordination types and one mild imbalance type in the nine divisions. The plain divisions Ⅰ and Ⅳ where the typical black soil belt is located are of high coupling coordination type, and the eco–geological conditions are good. Mountain divisions Ⅲ, Ⅶ and plain division Ⅴ are of moderate coupling coordination type. These areas have complex geological and geomorphological conditions or sensitive and fragile ecosystems. Mountain divisions Ⅱ and Ⅷ and plateau division Ⅸ are low coupling coordination types, of which Changbai Mountain is of geology–geomorphology lag type, the southern GKM is of simultaneous type and Hulunbuir Plateau is of ecology lag type. Mountain division Ⅵ is a geology–geomorphology lag type of mild imbalance.

The significant variation trend of NDVI in Northeast China from 2001 to 2020 is analysed by the Sen+MK method. The overall performance of NDVI in Northeast China shows a positive trend. For the divisions Ⅲ, Ⅴ, Ⅰ, Ⅱ and Ⅶ with significant increasing trend, the coupling coordination characteristics of geology–geomorphology and ecology may change with time, while the divisions Ⅳ, Ⅵ, Ⅷ and Ⅸ with significant decreasing trend of NDVI are mainly in the provincial capital cities, urban agglomeration in central and southern Liaoning Province and the southern end of the GKM. Combined with the characteristics of coupling coordination, these areas should strengthen ecological management and ecological construction.

## Supporting information

S1 Table Eco–geological characteristics of each division in Northeast China. (DOCX)

S1 File The algorithms of entropy weight and game theory. (DOCX)
