# Supplementary material for: Coupling coordination relationship between geology–geomorphology and ecology in Northeast China
Source: PLoS One. 2022 Apr 7;17(4):e0266392. doi: 10.1371/journal.pone.0266392 (PMC8989230; doi:10.1371/journal.pone.0266392)
Supplement: S1 Table — (DOCX) [file pone.0266392.s001.docx]

**S1 Table. Eco–geological characteristics of each division in Northeast China.**

| **Division NO. and Name** | **Eco**–**geological Characteristics** |
| --- | --- |
| Ⅰ  Sanjiang Plain | The main body is the Sanjiang Plain, which belongs to Jiamusi block, Wandashan junction zone and Xingkai block of Tianshan–Xingmeng orogenic system. It is a depression belt in the north of the second uplift belt of Neocathaysian tectonic system, belonging to Tongjiang inland fault depression in geotectonics. The landforms are plain with altitude of 40–90 m and low mountains and hills with altitude below 500 m. It has a temperate humid climate. The parent rocks in plain area are mainly quaternary alluvial deposits, part of alluvial lacustrine deposits and swamp deposits, and there are Mesozoic metamorphic rocks, granitoids and diorites in Wandashan area. Wetland and farmland are widely distributed, agriculture is developed. The vegetation types include meadow, swamp and cultivated plants in plain area and coniferous and broad–leaved mixed forest in low mountain and hilly area. Problems such as freeze–thaw disasters, wetland degradation and water Erosion desertification exist. |
| Ⅱ  The Changbai Mountains | The main body is the Changbai Mountains, which belongs to Xiaoxing'anling–Zhangguangcailing magmatic arc, Jiamusi block, Xingkai block, Baoerhantu–Wenduermiao arc basin system of Tianshan–Xingmeng orogenic system, and Jiaoliao block of North China block. The landforms are mainly composed of 500–1100 m middle and low mountains and hills. It has a humid climate of middle temperate and warm temperate. The lithology mainly includes granite, diorite, Quaternary volcanic accumulation, basalt, Archean and Proterozoic metamorphic rocks, and Quaternary glacial water accumulation. The vegetation types mainly include coniferous and broad–leaved mixed forest, deciduous broad–leaved forest and cultivated plants. It is an important ecological barrier in Northeast Asia. Problems such as water erosion desertification, landslide, geological disaster in mining areas, biodiversity protection and destruction of volcanic remains and native vegetation exist. |
| Ⅲ  The LKM | The main body is the Lesser Khingan Mountains (LKM), which belongs to Xiaoxing'anling–Zhangguangcailing magmatic arc and Greater Khingan Mountains (GKM) arc basin system of Tianshan–Xingmeng orogenic system. The landforms are mainly 500–1300 m low mountains, hills and lava platform. It has a temperate semi–humid and semi–arid climate. The lithology mainly includes granite, Mesozoic and Proterozoic metamorphic rocks, Cenozoic sandstone, Quaternary volcanic deposits, with a small amount of diorite. The forest is widely distributed in the area. The vegetation types mainly include coniferous and broad–leaved mixed forest and broad–leaved mixed forest. Problems such as water erosion desertification, frozen soil disaster, freeze–thaw debris flow exist. |
| Ⅳ  Eastern Songliao Plain | The main body is the eastern Songliao Plain, which belongs to Xiaoxing'anling–Zhangguangcailing magmatic arc and Songliao fault basin, and the southern end belongs to Jiaoliao block. The main landforms are sandy lacustrine plain and undulate platform below 500 m above sea level. It has a mid–temperate humid and semi–humid climate. The lithology mainly includes quaternary proluvial and alluvium, and granite and sedimentary rock in the north. The land cover types mainly include meadow grassland, forest grassland, farmland and city. As a typical black soil distribution area, problems such as water erosion desertification, soil fertility decline, land degradation, urban groundwater level decline occur. |
| Ⅴ  Central and western Songliao Plain | The main body is the central and western Songliao Plain, which belongs to the Songliao block of Tianshan–Xingmeng orogenic system, Xiaoxing'anling–Zhangguangcailing magmatic arc, Suolunshan– Xilamulun junction zone, Baoerhantu–Wenduermiao arc basin system, and Jiaoliao block of North China block. The landforms are plain with an altitude of 200–250 m. It has a humid, semi–humid and semi–arid climate of middle temperate. The lithology is mainly Quaternary, including Pleistocene alluvial lacustrine deposits, Holocene Aeolian, lacustrine, alluvial proluvial and alluvial deposits. The vegetation types mainly include temperate grassland, farmland, wetland and sandy land. Problems such as grassland degradation, sandy desertification, soil salinization and groundwater level decline exist. |
| Ⅵ  Western Liaoning Mountainous | This division is mainly located in upper reaches of the West Liao River and the western Liaoning Mountainous. It belongs to Baoerhantu–Wenduermiao arc basin system of Tianshan–Xingmeng orogenic system, Jiaoliao block, Jinji block and Daqingshan–northern Hebei ancient arc basin system of North China block. In the south, there are low mountains and hills below 1000 m above sea level, and in the north, there are denudation platforms. It has a humid, semi–humid and semi–arid climate of middle temperate and warm temperate zone. The lithology mainly includes Mesozoic sedimentary rocks, a small amount of granite, and Quaternary alluvial proluvial deposits along the coast. The main vegetation types include farmland, grassland, coniferous and broad–leaved mixed forest and urban. Problems such as aeolian sandy desertification and water erosion desertification occur . |
| Ⅶ  The central and northern GKM | The main part of this division is the GKM, which belongs to the GKM arc basin system and the Suolunshan–Xilamulun junction zone of the Tianshan–Xingmeng orogenic system. The landforms are mainly middle and low mountains of 500–1300 m. It has a temperate semi–humid and semi–arid climate. The lithology is dominated by Mesozoic metamorphic rocks and granitoids, intercalated with Quaternary volcanic deposits. The area is an important ecological barrier because of the wide distribution of virgin forest, the development of forest swamp and wetland. Problems such as freeze–thaw disaster, swamping and potential water erosion desertification exist. |
| Ⅷ  The southern GKM | The main body is the southern end of the GKM, belonging to the Zhalantun–Duobaoshan island arc, Erlian–Hegenshan ophiolitic melange belt, Xilinhot magmatic arc and the Suolunshan–Xilamulun junction belt of the GKM arc basin system. The landforms are low and medium mountains with bedrock at an altitude of 500–1300 m. The lithology mainly includes variegated acid pyroclastic rocks, acid lava with intermediate acid pyroclastic rocks and pyroclastic sedimentary rocks. It has a mid–temperate semi–humid and semi–arid climate. The main vegetation type is the falling forest grassland. Problems such as forest degradation and potential sandy desertification exist. |
| Ⅸ  Hulunbuir Plateau | The main body is Hulunbuir Plateau, belonging to Hailaer–Huma back arc basin of the GKM arc basin system of Tianshan–Xingmeng orogenic system. The landforms are high platform and plain with an altitude of 500–1000 m. It has a mid–temperate semi–arid climate. The lithology is mainly composed of Holocene lacustrine deposits, alluvial deposits, Permian granite, mudstone, sandstone, glutenite of lower Cenozoic, acid volcanic lava and clastic rocks of Mesozoic. The main vegetation type is grassland. The grassland and animal husbandry is well developed in this division, and the mineral resources are rich. Problems such as grassland quality degradation, sandy desertification, salt desertification and freeze–thaw disasters exist. |
