# Supplementary material for: Coupling coordination relationship between geology–geomorphology and ecology in Northeast China
Source: PLoS One. 2022 Apr 7;17(4):e0266392. doi: 10.1371/journal.pone.0266392 (PMC8989230; doi:10.1371/journal.pone.0266392)
Supplement: S1 File — (DOCX) [file pone.0266392.s002.docx]

# S1 File. The algorithms of entropy weight and game theory.

## Entropy weight

The main formulas are shown in equations (1) to (4) [1].

| $p_{ij}=\frac{x_{ij}^{'}}{\sum_{i=1}^{n} x_{ij}^{'}}$ | (1) |
| --- | --- |
| $e_{j}=-k\sum_{i=1}^{n} p{}_{_{ij}}\ln(p_{ij})$ | (2) |
| $d_{j}=1-e_{j}$ | (3) |
| $w_{j}=\frac{d_{j}}{\sum_{j=1}^{m} d_{j}}$ | (4) |

where $p_{ij}$ is the proportion of the i–th division value under the j–th index in the indices; $x_{ij}^{'}$ is the standardised value of the j–th index of the i–th division; $e_{j}$ is the entropy of index j, and $e_{j}\geq0$; $k=1/\ln(n)>0$; $d_{j}$ is the information entropy redundancy (difference value), and $w_{j}$ is the weight of each index.

## Game theory

If *L* different weighting methods are used to weight the indices (in this study, *L* = 2), and the basic weight vector set $u_{k}=\left\{u_{k1},u_{k2},\cdots,u_{kn}\right\}$ ($k=1,2,\cdots,L$) is constructed, then any linear combination of *L* different vectors is expressed in equation (5).

| $u_{0}=\sum_{k=1}^{L} \beta_{k}u_{k}^{T}$ （$\beta_{k}>0$） | (5) |
| --- | --- |

where $u_{0}$ is the combination weight, *k* is the superposition coefficient, $u_{k}$ is the weight vector of the *k*–th method, and $\beta_{k}$ is the linear combination coefficient.

Combination vectors are optimised to minimise the sum of the range differences between the optimal weight vector $u_{0}$ and other $u_{k}$. The optimal first derivative condition is obtained using the differential property of matrix which can be transformed into a system of linear equations. The formulas are shown in equations (6) to (8).

| $\min=\left\Vert\sum_{m}^{L} \beta_{i}u_{m}^{T}-u_{i}^{T}\right\Vert_{2}$（$i=1,2,\cdots,L$） | (6) |
| --- | --- |
| $\sum_{m}^{n}\beta_{m}u_{i}u_{m}^{T}=u_{i}^{T}u_{i}$ | (7) |
| $\left[\begin{matrix} u_{1}u_{1}^{T} & u_{1}u_{2}^{T} & \cdots& u_{1}u_{L}^{T} \\ u_{2}u_{1}^{T} & u_{2}u_{2}^{T} & \cdots& u_{2}u_{L}^{T} \\ \vdots& \vdots& \cdots& \vdots\\ u_{L}u_{1}^{T} & u_{L}u_{2}^{T} & \cdots& u_{L}u_{L}^{T} \end{matrix}\right]\left[\begin{matrix} \beta_{1} \\ \beta_{2} \\ \vdots\\ \beta_{L} \end{matrix}\right]=\left[\begin{matrix} u_{1}u_{1}^{T} \\ u_{2}u_{2}^{T} \\ \vdots\\ u_{L}u_{L}^{T} \end{matrix}\right]$ | (8) |

The result is $\text{（}\beta_{1}\text{，}\beta_{2}\text{，}\cdots\text{，}\beta_{L}\text{）}$. The linear coefficient $\beta_{k}^{*}$ is obtained through normalisation as equation (9). Then, the calculation method of the final comprehensive weight combination vector is shown in equation (10) [2].

| $\beta_{k}^{*}=\frac{\left\vert\beta_{k}\right\vert}{\sum_{k=1}^{L} \left\vert\beta_{k}\right\vert}$ | (9) |
| --- | --- |
| $u_{k}^{*}=\sum_{k}^{L} \beta_{k}^{*}u_{k}^{T}$ | (10) |

# References

1. Cai ZY, Li WM, Cao SX. Driving factors for coordinating urbanization with conservation of the ecological environment in China. Ambio. 2021; 50: 1269-1280. doi:10.1007/s13280-020-01458-x.
2. Liu Y, Hu YC, Hu YM, Gao YQ, Liu ZY. Water quality characteristics and assessment of Yongding New River by improved comprehensive water quality identification index based on game theory. J Environ Sci-China. 2021; 104: 40-52. doi:10.1016/j.jes.2020.10.021.
